# Examination of well ordered nanonetwork materials by real- and reciprocal-space imaging

**DOI:** 10.1107/S2052252518018389

**Published:** 2019-02-15

**Authors:** Po-Ting Chiu, Yu-Cheng Chien, Prokopios Georgopanos, Ya-Sen Sun, Apostolos Avgeropoulos, Rong-Ming Ho

**Affiliations:** a Department of Chemical Engineering, National Tsing Hua University, No. 101, Section 2, Kuang-Fu Road, Hsinchu 30013, Taiwan; b Department of Materials Science and Engineering, University of Ioannina, University Campus, Ioannina 45110, Greece; c Institute of Polymer Research, Helmholtz-Zentrum Geesthacht, Max-Planck-Strasse 1, Geesthacht 21502, Germany; d Department of Chemical and Materials Engineering, National Central University, No. 300 Zhongda Road, Taoyuan 32001, Taiwan

**Keywords:** nanonetworks, gyroid structures, gold nanoparticles, electroless plating, block copolymers, form factors, structure factors, materials science, nanoscience, SAXS, inorganic porous solids, time-resolved crystallography

## Abstract

The development of well ordered nanonetwork materials (in particular gyroid-structured materials) has been investigated using a block-copolymer template for templated electroless plating as an example system for the examination of network formation using X-ray scattering. By taking advantage of the nucleation and growth mechanism of templated electroless plating, gyroid-structured Au was successfully fabricated through the development of Au nanoparticles, then tripods and branched tripods, and finally an ordered network. Each stage in the development of the network phase could then be examined by combining real-space transmission electron microscopy observations with reciprocal-space small-angle X-ray scattering results. The fingerprint scattering profile of the building block for the network (*i.e.* the tripod of the gyroid) could be well fitted with the form factor of an effective sphere, and the diffraction results from the ordered network could thus be reasonably addressed. As a result, the examination of well ordered network materials can be simplified as the scattering from the form factor of a sphere convoluted with the nodes of its structure factor, providing a facile method of identifying the network phases from X-ray scattering data.

## Abstract 2

The development of well ordered nanonetwork materials from basic building blocks to the bulk phase is examined by simplifying the scattering from the form factor of a sphere convoluted with the nodes of the structure factor, providing a simple methodology for studying the formation of well ordered nanonetwork materials.

## 1. Introduction   

Well ordered network structures from the self-assembly of biomaterials (Saranathan *et al.*, 2010 ▸; Wilts *et al.*, 2012 ▸; Yu *et al.*, 2013 ▸) and synthetic materials (Luzzati & Spegt, 1967 ▸; Mariani *et al.*, 1988 ▸; Ichikawa *et al.*, 2007 ▸; Zeng *et al.*, 2005 ▸; Seddon & Templer, 1993 ▸; Kresge *et al.*, 1992 ▸; Hajduk *et al.*, 1994 ▸; Takenaka *et al.*, 2007 ▸) have received intense attention in recent decades. Owing to their complex material architecture and surface geometry, nanonetwork structured materials have been used in a wide variety of applications in solar cells (Crossland *et al.*, 2009 ▸), supercapacitors (Crossland *et al.*, 2009 ▸), photonic crystals (Maldovan *et al.*, 2002 ▸; Hur *et al.*, 2011 ▸; Hsueh *et al.*, 2014 ▸) and plasmonic metamaterials (Hur *et al.*, 2011 ▸; Hsueh *et al.*, 2013 ▸).

Such materials can be characterized by small-angle X-ray scattering (SAXS) and transmission electron microscopy (TEM) (Hajduk *et al.*, 1994 ▸; Epps *et al.*, 2004 ▸). Diffraction results can provide detailed information about the symmetry of a structure, so the space group of a network structure can be identified by its structural skeleton. However, because of their complex network structure, it is challenging to construct the real-space symmetry from a reciprocal-space 2D diffraction pattern, even with 3D diffractometry (Miao *et al.*, 2002 ▸).

Block copolymers (BCPs) are a well known class of self-assembled systems because of their ability to self-assemble into a variety of nanostructured phases through microphase separation (Bates & Fredrickson, 1999 ▸; Hajduk *et al.*, 1994 ▸; Epps *et al.*, 2004 ▸; Takenaka *et al.*, 2007 ▸; Chu *et al.*, 2015 ▸). Self-consistent field theory reveals that the texture of the network in BCP systems is much more complex than in other self-assembled systems due to packing frustration of the long polymer chains (Matsen & Schick, 1994 ▸); thus, the genuine textures of such networks are still under debate. Existing studies of well ordered networks have generally focused on the characterization of the network struts as a whole. Detailed analyses of the shape and texture of the building blocks in a network within their unit cells (*i.e.* the form factor of the building block, such as a tripod for a gyroid phase and a tetrapod for a diamond phase) remain unidentified. Since every phase has its own building block, such as atoms for metallic materials and giant molecules for nanostructured phases, it is essential to acquire detailed information at every level, all the way through from the building block to the final well ordered network structure. Therefore, establishing a facile method for the identification of the developing nanonetwork through X-ray scattering approaches is essential. It is especially appealing to build up a methodology for the examination of the structural development from the building block to the complete network phase.

**Figure 1:**
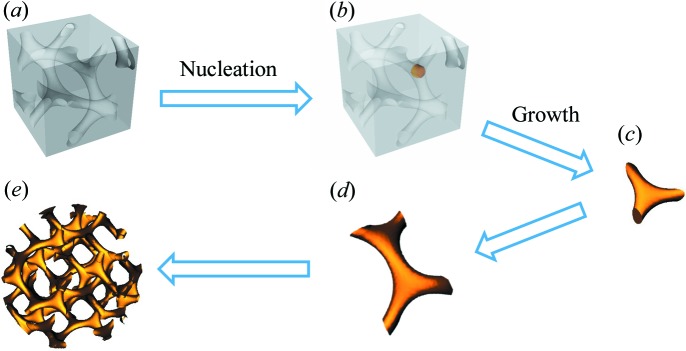
A schematic illustration of the development of well ordered network Au from templated electroless plating using gyroid-structured PS–PDMS as a template. (*a*) The nanoporous PS template, formed from self-assembled PS–PDMS followed by hydrogen fluoride acid etching of the PDMS. (*b*) Nucleation of the Au nanoparticle by templated electroless plating. (*c*) The growth of tripod Au from the Au nanoparticle. (d) Branching of the tripod Au. (*e*) The formation of gyroid-structured Au.

Herein, we aim to examine the formation of self-assembled nanonetwork materials from the building block to the bulk phase using a BCP as an example system. Templated electro­less plating was introduced using polystyrene–*block*-poly(dimethylsiloxane) (PS–PDMS) BCPs with nanonetwork phases (Politakos *et al.*, 2009 ▸; Lo *et al.*, 2013 ▸), in particular a gyroid phase, as a template. As illustrated in Fig. 1 ▸, the branching of the tripod (the building block) through the nucleation and growth mechanism is accomplished within the template. Among the other templated technologies, sol–gel synthesis cannot achieve exactly a segment of a network but only a whole (Hsueh *et al.*, 2010 ▸; Lin *et al.*, 2017 ▸; Hsueh & Ho, 2012 ▸), and electrochemical deposition needs a conductive substrate to pass the electric current for the development of a complete network (Scherer *et al.*, 2012 ▸). By contrast, in electro­less plating it is possible to develop network formation from a building block, and the growth of the tripod, from the branching of a reduced nanoparticle as a nucleus to the complete network-structured phase, can be tuned precisely (Hsueh *et al.*, 2013 ▸). Consequently, the developing PS/Au nanohybrids in the bulk template at different stages can be examined by real-space TEM imaging for comparison with reciprocal-space imaging from SAXS. A facile method is provided here for the examination of the intended network formation as a well ordered phase, and a simple approach is proposed for the modelling of scattering results for network-structured phases.

## 2. Experimental   

### 2.1. Samples   

Gyroid-structured Au samples were synthesized by templated electroless plating as described in the previous study (Hsueh *et al.*, 2015 ▸). At the nucleation stage, Au nanoparticles were reduced from Au ions (Au^3+^) in the presence of reducing agents (hydrazinium hydroxide, N_2_H_5_OH), giving randomly seeded Au nanoparticles within the nanoporous PS polymer template. This PS template resulted from the self-assembly of PS–PDMS, followed by hydrogen fluoride (HF) etching to remove the PDMS block. In the templated growth process, weak reducing agents (diethanolamine, DEA) were then used to ensure the formation of nanostructured metal *via* branching, giving rise to the formation of the tripod building block and the textures of interest.

### 2.2. Transmission electron microscopy   

Bright-field TEM images were obtained using mass–thickness contrast with a JEOL JEM-2100 LaB_6_ transmission electron microscope operated at an accelerating voltage of 200 kV. The bulk samples of PS–PDMS were sectioned by ultra-cryomicrotomy (−160°C) using a Reichert Ultracut microtome to a thickness of 150 nm. Alternatively, bulk samples of PS/Au were sectioned at room temperature using a Leica Ultra-microtome to a thickness of 100 nm. Afterwards, the microsections were collected on copper grids (200 mesh). Staining is not necessary, since the electron densities of PDMS and Au are large enough compared with PS to create sufficient contrast.

### 2.3. Small-angle X-ray scattering   

Small-angle X-ray scattering (SAXS) experiments were conducted on the synchrotron X-ray beamline BL23A at the National Synchrotron Radiation Research Center (NSRRC) in Hsinchu, Taiwan. The wavelength of the X-ray beam was 0.155 nm. A MAR CCD X-ray detector (MAR USA) was used to collect the 2D SAXS patterns. A 1D linear profile was obtained by integration of the 2D pattern. The scattering angle of the SAXS pattern was calibrated using silver behenate, with the first-order scattering vector q* = 1.076 nm^−1^ (q* = 4λ^−1^sin θ, where 2θ is the scattering angle). The bulk samples were heated at 140°C for 5 min before collecting data to preclude any signal from the template. We also used *in situ* SAXS to trace the collapse of the template. For this, the bulk samples were heated up to 140°C at a heating rate of 8°C min^−1^.

### 2.4. Analysis of SAXS data   

The scattering profiles are proportional to the product of the form and structure factors. The acquired scattering results were analysed using a polydispersed sphere, cylinder, disk or cube as the form factor and a gyroid texture as the structure factor (Förster *et al.*, 2010 ▸). A log–normal distribution was introduced into the fit to provide polydispersity in the radii of the sphere, cylinder or disk and in the length of the cube. Consequently, the parameters of each model could be extracted from the fit of the form factor with the structure factor set as 1. These parameters were then used to simulate scattering profiles of the gyroid-structure network phase by combining the form and structure factors.

## 3. Results and discussion   

### 3.1. Morphological evolution of a gyroid-structured network   

**Figure 2:**
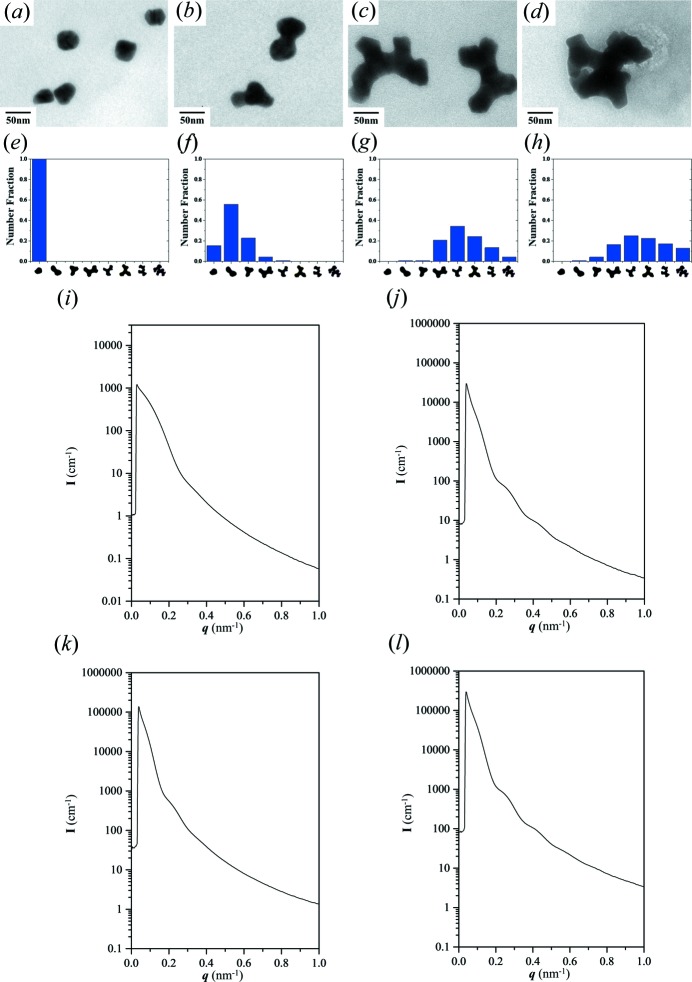
TEM micrographs, histograms of shape distribution and one-dimensional SAXS profiles, respectively, of gyroid Au nanoparticles in the early stages of templated electroless plating at (*a*), (*e*), (*i*) 5 h, (*b*), (*f*), (*j*) 33 h, (*c*), (*g*), (*k*) 39 h and (*d*), (*h*), (*l*) 45 h. The statistical results were analysed from over 300 particles.

As shown in Figs. 2 ▸(*a*)–2 ▸(*d*), the morphological development from dot to tripod to branched-tripod Au in the PS matrix can be directly visualized by TEM observations using self-assembled PS–PDMS as a template for the growth of reduced Au to fabricate PS/Au nanohybrids. In the early stages [as shown in Fig. 2 ▸(*a*) for 5 h reduction], the reduction of Au ions gives rise to the formation of nuclei for the development of tripod texture. When Au starts to grow from these nuclei, the nanochannels will be gradually filled up to form embryos of the gyroid texture [Fig. 2 ▸(*b*)]. As time goes on, each embryo can gradually transform into the basic building block of the gyroid texture, namely, the development of a tripod with one node. Subsequently, branched tripods with at least two nodes of the network can be clearly identified [Fig. 2 ▸(*c*)]. After prolonged reduction and growth (*e.g.* 45 h), these Au nano-objects can develop into a well ordered network structure like a unit cell [Fig. 2 ▸(*d*)]. Statistical analysis of the real-space TEM observations can be used to analyse the controlled growth of reduced Au into a connective network. A clear picture of building up the gyroid texture can be seen from the distribution of the developing morphology initiated from the spherical nucleus [Fig. 2 ▸(*e*)] to the embryo of the tripod and the branched texture [Fig. 2 ▸(*f*)]. The degree of branching can easily be distinguished from the number of nodes in the network. For instance, after 39 h of reduction, a slightly branched texture predominantly consisting of more than three nodes is found [Fig. 2 ▸(*g*)]. After continued reduction, further growth of the reduced Au is observed, which leads to the morphological formation of more than five nodes from branching [Fig. 2 ▸(*h*)].

As a complement to real-space TEM imaging, X-ray scattering experiments were carried out to provide macroscopic examination of the developing network. As shown in Fig. 2 ▸(*i*), the resultant fingerprint scattering profile in the corresponding one-dimensional SAXS results is attributed to the formation of an Au nucleus as a sphere-like texture. Most interestingly, the corresponding one-dimensional SAXS results for the developing Au from an embryo to a tripod, and then to a branched tripod, all appear as fingerprint scattering profiles [Figs. 2 ▸(*j*)–2 ▸(*l*)] in which all the patterns are similar except for variations in intensity. We speculate that this is a signature of the presence of duplicate tripod texture from the basic building block with the gradual increase in volume of Au as the growth time extends (see below for the reasons for this). Generally, when the radius (volume) of a particle increases, the fingerprint of its form factor will shift to the left. However, the situation in this case is obviously different to the scattering from common nanoparticles. As a result, we aim to examine such unique scattering results for the growing tripod using the following single equation:
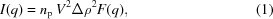
where *q* is the scattering vector, *n*_p_ is the particle density of the scatterer, *V* is the volume of the scatterer, Δρ is the scattering length density contrast between PS and Au, and *F*(*q*) is the form factor of the gyroid building block. We can exclude thickness effects during signal subtraction, and so the variation in intensity can be attributed to either *n*_p_ or *V*, since Δρ is constant. Because the particle density is determined at the nucleation stage, it is thus regarded as a constant; hence, the volume of the scatterer gives rise to the variation in intensity. It should be noted that a change in *V* is not accompanied by a variation in *F*(*q*), which might give changes in scattering. From the viewpoint of the building block, the whole structure consists of basic elements which contribute the same scattering profile. Those tripods should be duplicated and placed at specific positions during the process of templated electroless plating for the construction of a gyroid phase from the branching of the tripod as a network texture. There is no structure factor involved in the SAXS profiles at this stage. We conjecture that a possible reason for the fingerprint profile could be attributed to the small size of the branched tripods that make up the gyroid texture as a phase with complete *I*4_1_32 symmetry. Also, there is no actual interparticle distance in the branched tripod for network formation due to the continuity of the developing tripod for the final network texture, *i.e.* the scattering contrast from the tripods is not recognized as giving significant diffraction before it develops as a whole with structural symmetry. Each of the above observations leads to the conclusion that it is the building block of the gyroid after formation of embryos that contributes these unique scattering results. Hence, we conclude that the fingerprint scattering profile gives the characteristics for the form factor of the gyroid (*i.e.* the tripod).

**Figure 3:**
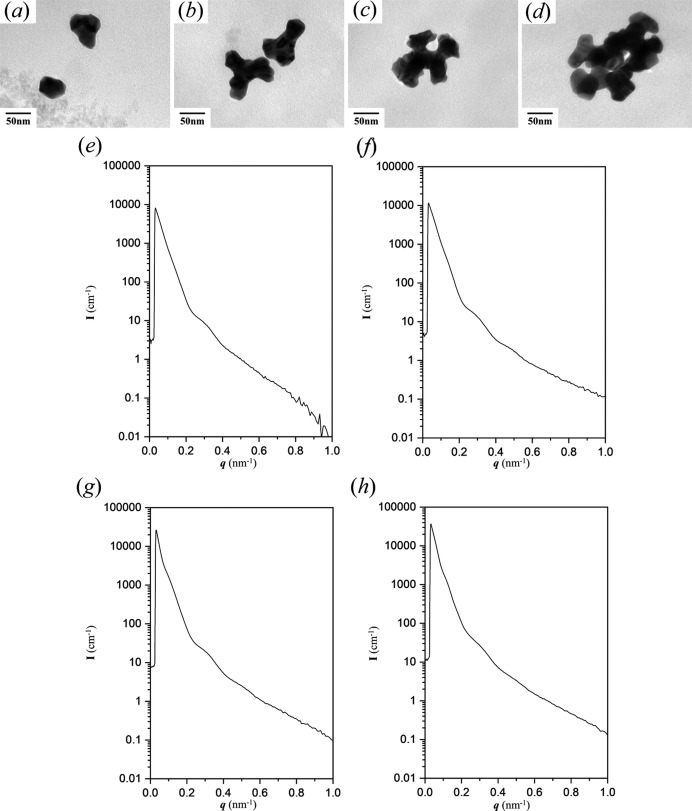
TEM images and 1D SAXS profiles, respectively, of gyroid Au nanoparticles of small size in the early stages of templated electroless plating at (*a*), (*e*) 12 h, (*b*), (*f*) 18 h, (*c*), (*g*) 24 h and (*d*), (*h*) 36 h.

A template with a small pore size was fabricated for templated electroless plating to obtain further evidence to confirm the above speculation. As shown in Figs. 3 ▸(*a*)–3 ▸(*d*), a tripod and a branched tripod within one unit cell of the gyroid can be formed within 24 h, and it is obvious that a smaller feature size of Au tripod can be formed if a template with a low molecular weight is used. Moreover, in contrast with the tripods formed from PS–PDMS with a large molecular weight, it is much easier and faster to fill a relatively narrow nanochannel, and so the required time for complete growth of gyroid-structured Au is shorter. Thus, we have primarily examined and compared the suggested characteristics of the scattering results from Au in a small-sized gyroid texture with the results from a large-sized gyroid texture. Note that the real-space images for the morphological evolution of branched Au of small size [Figs. 3 ▸(*a*)–3 ▸(*d*)] are similar to the previous ones in Fig. 2 ▸. Figs. 3 ▸(*e*)–3 ▸(*h*) show the corresponding one-dimensional SAXS profiles of the reduced Au nano-objects in Figs. 3 ▸(*a*)–3 ▸(*d*), and the fingerprint profiles can be clearly observed. The intensity in the high-*q* region is lower in Fig. 3 ▸(*e*) due to the significant background intensity around 0.1 cm^−1^ (see Fig. S2A in the supporting information); note that the scattering results are not reliable if the intensity is below this value. It is apparent that, with the growth of reduced Au nano-objects, the trend of variation in the scattering profiles is similar to that observed for larger dimensions, *i.e.* as long as the fabricated templates give similar textures, the fingerprint profiles remain consistent regardless of the growing size of the reduced Au. The only notable difference is the variation in intensity due to the increasing volume of branching Au from the embryo of the gyroid building block.

### 3.2. Fitting of the gyroid form factor with an effective sphere   

For a true characterization of the scattering results from the network phase, it is necessary to resolve the scattering contributions from both the form factor and the structure factor. Note that the form factor is referred to as the building element for the development of the structure as a phase. In general, SAXS profiles are fitted based on the shape of the building block and the corresponding size. It is noted that the fit of the tripod texture is obviously not a sphere and that it is difficult to provide a fitting model for such a complicated texture with various thicknesses of the framework. As this study shows, there is no significant variation in the scattering profile with fingerprint-like texture at specific *q* positions; the increment in the scattering intensity is affected by the growth of the reduced Au as it starts branching to give the tripod texture, followed by the formation of branched Au for networking from the initial tripod. A simple approach is proposed to fit the scattering results from the tripod texture using an effective sphere shape. By fitting the tripod with an effective sphere, the information hidden in the SAXS profile may be deciphered. The spherical texture form factor is given by 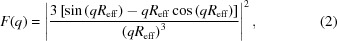
where *q* is the scattering vector and *R*_eff_ is the radius of the effective sphere.

**Figure 4:**
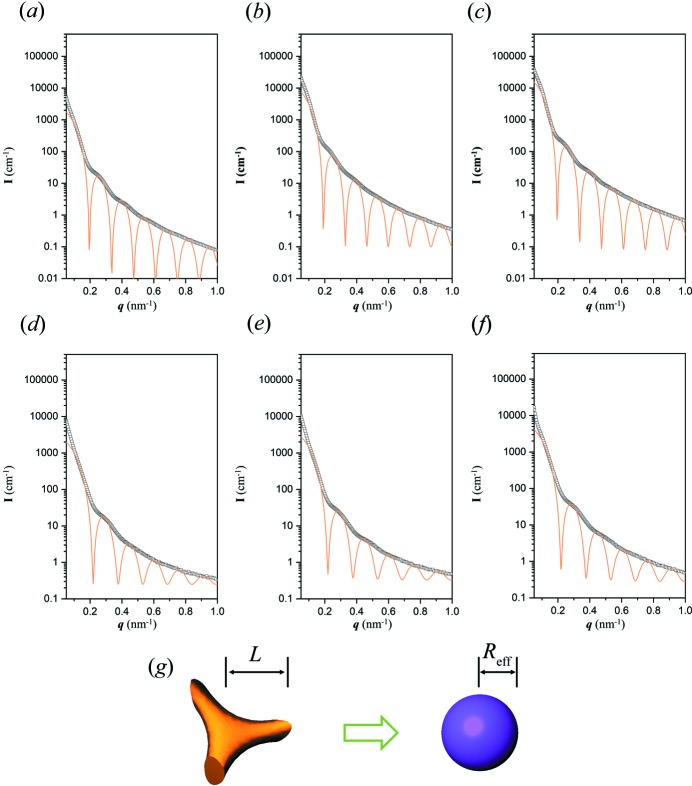
Fitting of SAXS profiles of reduced Au nanoparticles acquired from (*a*), (*b*), (*c*) a large template and (*d*), (*e*), (*f*) a small template, both with a form factor of a sphere, in the early stages of the growth of Au nano-objects from templated electroless plating at (*a*) 33 h, (*b*) 39 h, (*c*) 45 h*r*, (*d*) 18 h, (*e*) 24 h and (*f*) 36 h. The fitting radii used were 23.0 and 20.5 nm, respectively, with the standard deviation set as 0.01. (*g*) A schematic illustration of the effective sphere model.

A consistent texture (the tripod) was acquired at 33 h, which results in a unique fingerprint scattering profile as the form factor of the gyroid building block; as shown in Figs. 4 ▸(*a*)–4 ▸(*c*), the fit is well addressed by a sphere with an effective radius of 23.0 nm. Among all three profiles of reduced Au with large dimensions, the only difference between the fits is the intensity, which increases with the growth of the reduced Au from branching. The same approach was applied to Au with small dimensions to justify the feasibility of the fit, and similar results were obtained [Figs. 4 ▸(*d*)–4 ▸(*f*)]. The hump positions in the scattering profiles can be fitted using a value of *R*_eff_ = 20.5 nm, which is consistently smaller than the dimensions of the large-sized Au. For systematic examination, in addition to the effective sphere model, different models including disk, cube and cylinder were used for the fitting of the scattering profiles (Fig. S3). In contrast with the effective sphere model, the disk model (Fig. S3*A*) can be excluded since the intensity variation is clearly disparate, whereas the cube model (Fig. S3*B*) and cylinder model (Fig. S3*C*) show fitting results similar to the effective sphere model. However, the shape of a cube is far from a tripod. For the fit using a cylinder, it is only feasible to have the length of the cylinder reach the unit-cell dimension, which is obviously unrealistic. The effective sphere therefore serves as a succinct model with acceptable fitting results, reasonable modelling and appropriate physical meaning. As the modules of the gyroid network, the tripods are connected to each other and positioned on specific spots, which are known as the nodes of the gyroid. According to the coordinates of the nodes with the lattice parameter *a*, the distance between two adjacent nodes is 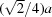, calculated to be 54.7 and 47.8 nm for the large and small gyroid-textured Au, respectively. The radii of 23 and 20.5 nm are just smaller than the half-distance between two nodes (*i.e.* the size of a tripod) in both cases [Fig. 4 ▸(*g*)]. Since the form factor of a sphere can be regarded as a simplified gyroid form factor, the effective sphere is approximately equivalent to the building block of the gyroid. Moreover, the size of the effective sphere is directly related to the size of the gyroid struts and has the same scale as a tripod, suggesting that the building block of the gyroid is a tripod. As the gyroid strut has different thicknesses along the skeleton, being thickest at the nodes and thinnest at the mid-point between two nodes, it gives a possible explanation for the similarity between the tripod and the sphere. We thus believe that this kind of substitution should provide a rational method to simplify the building blocks of network structures as a sphere to reduce the complexity of the form factor for the corresponding analysis of simulation of SAXS results from reciprocal-space imaging.

### 3.3. Development of a gyroid network from a tripod building block   

With the form factor of the tripod as an effective sphere, the development of the gyroid from the initial branching to the network structure can be examined based on the scattering results from the growth of reduced Au. Accordingly, the scattering results of the growing gyroid can be fitted through the convolution of the form factor (the effective sphere) and structure factor (the space-group symmetry). Note that a double gyroid with 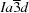 symmetry is constructed by two single gyroid struts with *I*4_1_32 symmetry, which is a subgroup of 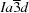. Following the growth of reduced Au after branching of the tripod, it is expected that structural symmetry will develop, giving the scattering profile as gyroid-structured Au with eight nodes within a unit cell to accomplish *I*4_1_32 symmetry. To fulfil this required development, the branching tripod and the developing network of reduced Au should be larger than the unit cell.

**Figure 5:**
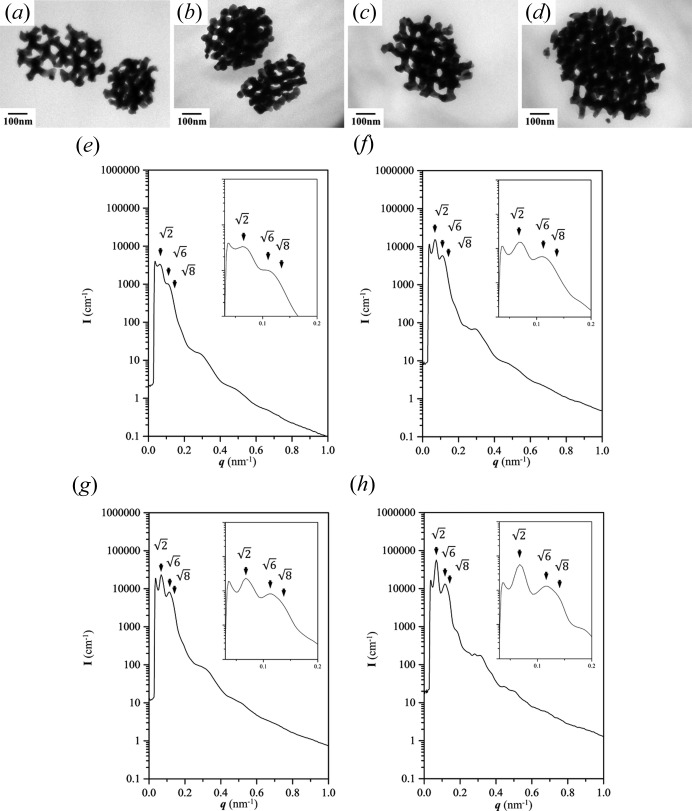
TEM images and 1D SAXS profiles, respectively, of gyroid-structured Au of small dimensions in the late stages of templated electroless plating at (*a*), (*e*) 2 days, (*b*), (*f*) 4 days, (*c*), (*g*) 6 days and (*d*), (*h*) 7 days. The characteristic gyroid reflections with relative *q* values of 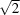, 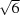 and 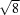 are marked by arrow heads. The insets are enlargements of the low-*q* regions.

To demonstrate the development from a branching tripod to a gyroid network, templated electroless plating for one week of growth was examined by TEM observations for real-space imaging. However, a template with a large-sized gyroid texture might take a much longer time to grow the required network, as larger unit cells take longer to acquire sharp reflections (Fig. S5). In contrast with Au of large dimensions, as shown in Fig. 5 ▸, the diameter of the struts fabricated could be reduced by using a template with a smaller lattice constant. Reduced Au with a size of approximately 200 nm can be observed after 2 d of growth [Fig. 5 ▸(*a*)], which is about one and a half unit cells. With further growth, the size of the network Au is larger [Fig. 5 ▸(*b*)]. Fig. 5 ▸(*c*) shows that, after 6 d of growth, the size of the resulting networked Au could reach approximately 500 nm, giving a size of over four unit cells for diffraction. Subsequently, the size of reduced Au could be as big as 800 nm, which is approximately six unit cells [Fig. 5 ▸(*d*)]. Owing to the smaller template, the number of repeat units increases much more quickly during electroless plating, thus making it easier to obtain duplicated texture. Consequently, the SAXS profiles [Figs. 5 ▸(*e*)–5 ▸(*h*)] show clear characteristic gyroid reflections with relative *q* values of 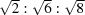. Moreover, high-*q* reflections can also be observed, demonstrating the development of long-range order for reduced Au with a gyroid-structured network. The intensity of the reflections increases with the increase in the number of repeat units in the growth of Au, and those reflections become sharper while the full width at half-maximum (FWHM) reduces, which is in line with the results expected from the Scherrer equation. Accordingly, the scattering and diffraction results are in harmony with the suggested structural development of reduced Au from a branching tripod to a gyroid network through templated electroless plating.

### 3.4. Simplification of network structure for scattering   

**Figure 6:**
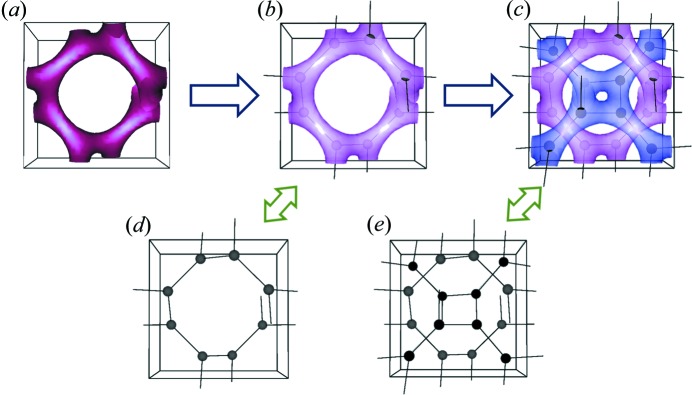
Schematic illustrations of (*a*) the unit cell of a single gyroid, (*b*) the nodes of a single gyroid and (*c*) the nodes of a double gyroid, with the corresponding simplified models (*d*) and (*e*) in the respective unit cells. The effective spheres are featured as nodes for visualization. Lines are used as a guide to the eye.

By combining the building block with an effective sphere and the structural symmetry of node positions for a developing network phase, a simple model is proposed for the prediction of scattering results from a tripod and corresponding diffraction results with respect to the growing gyroid-structured network. As illustrated in Figs. 6 ▸(*a*) and 6 ▸(*b*), there are eight nodes in the unit cell of a single gyroid, at the centres of the constituent tripods as defined by Wyckoff positions. As revealed above, the scattering from a tripod can be simplified as an effective sphere, which is the building block of the gyroid based on the suggested model. Specifically, the gyroid building block can be replaced by an effective sphere. With the corresponding model as illustrated in Fig. 6 ▸(*d*), the ‘flesh’ on the network skeleton is neglected, greatly reducing the complexity, while the symmetry and handedness can be faithfully preserved. Note that the thickness of the flesh for a gyroid phase from self-assembly varies from place to place. Furthermore, a double gyroid phase is generally obtained from the self-assembly of molecules and supramolecules instead of a single gyroid for reasons of thermodynamic stability, by which a pair of right- and left-handed gyroid networks gives the most stable phase thermodynamically.

It is straightforward to test the suggested model for a double gyroid-structured template with good contrast from the air nanochannels without considering the scattering from the polymer chain configurations, and a single gyroid is the subgroup of a double gyroid that can easily apply this model from different network systems. As demonstrated in Figs. 6 ▸(*c*) and 6 ▸(*e*), the structure with a double gyroid of 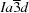 symmetry, where the frameworks with flesh (*i.e.* the form factor) are substituted by the effective sphere and the symmetry (*i.e.* the structure factor) is derived from its Wyckoff positions, can be simply defined based on the single gyroid texture.

**Figure 7:**
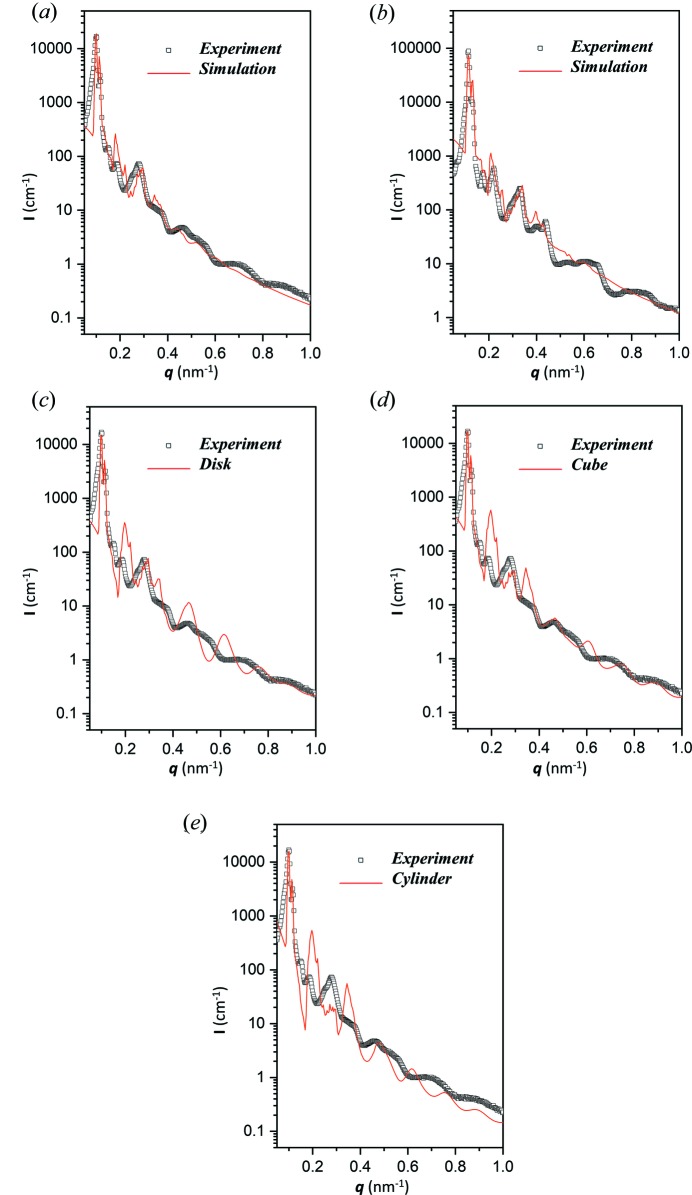
(*a*), (*b*) Simulations of the SAXS results of the nanoporous template with a double-gyroid texture fabricated from two PS–PDMS samples with different molecular weights using the form factor of the effective sphere and the structure factor of a double gyroid. (*c*)–(*e*) Simulations of SAXS results from the template with a large-sized gyroid texture using the form factors of (*c*) a disk, (*d*) a cube and (*e*) a cylinder.

A nanoporous PS with double gyroid-structured nanochannels was used to examine the feasibility of the proposed model. Figs. 7 ▸(*a*) and 7 ▸(*b*) show simulated SAXS profiles (solid red lines) for double gyroid-structured PS templates from self-assembled PS–PDMS with different molecular weights by convolution of the form factor of a sphere and the structure factor of a double gyroid for comparison with the experimental results (hollow grey squares). The form factor is the radius of the effective sphere for which two specific values of 23.0 and 20.5 nm were used for the predicted results with a homogeneous air core. A log–normal distribution with a standard deviation of 0.06 for the assumed radius was used to rationalize the simulated profiles. Note that a log–normal distributed radius is necessary for the continuity of the curve to approximate the gyroid form factor. The structure factor is the lattice parameter, for which two specific values of 154.7 and 135.2 nm were used based on the SAXS results. As shown in Fig. 7 ▸(*a*), both peak positions and FWHM can be properly addressed to give an approximate fit to the experimental results, with the maximum intensity of the simulated result set to fit the first peak of the experimental one. The intensity variations of the reflections from the double gyroid-structured template were simulated by simple convolution without any further or complicated assumptions required. Similar simulation results have been obtained which show clear reflections and consistent intensity variations [Fig. 7 ▸(*b*)] to the experimental one from the template with a small gyroid-structured texture.

To demonstrate the validity of the effective-sphere model, simulations with different form factors such as disk, cube and cylinder were also examined [Figs. 7 ▸(*c*)–7 ▸(*e*) and Fig. S5] based on the parameters obtained from the fitting of the gyroid form factor. In those cases, only a few reflections can reasonably match the scattering profiles of the templates, resulting in unsatisfactory mid- or high-*q* regions. Even though the cube and cylinder models show similar form factors to the effective-sphere model, the fitting results for the gyroid phase are not acceptable.

The simulation results thus provide a simple demonstration of the feasibility of using the form factor of a sphere to substitute for the gyroid form factor in the node positions of the gyroid texture to address the scattering profile from the periodic gyroid architecture. We infer that this idea could be reliably used in different well ordered network structures. Admittedly, the simulated results are not entirely congruent with the experimental ones for some reflections at high *q*, but this could be attributed to the difference between the fine structure in the network structure and the simplified form factor. Accordingly, this should be a facile method for the fitting of scattering results from a well ordered network structure by assuming the form factor to be an effective sphere. On the basis of the form factor examination reported in this study, it should be reasonable to apply this model to different network structures constructed by the connection of building blocks, such as a tetrapod for a single diamond network structure in the *Fd*3*m* space group and also a double diamond network structure in the *Pn*3*m* space group, as well as a hexapod for the plumber’s nightmare network structure in the 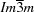 space group.

## 4. Summary and conclusions   

A new and simple methodology for the examination of the structural evolution of well ordered network texture such as a gyroid structure by reciprocal-space imaging (*i.e.* SAXS) has been proposed, and further demonstrated by real-space imaging (*i.e.* TEM). Au with a tripod texture was acquired by templated electroless plating, and then used as a building block for the examination of the gyroid form factor using X-ray scattering. The fingerprint scattering profile can be found and well fitted by an effective sphere with an invariant diameter, regardless of the degree of branching of the tripod. Accordingly, the form factor of the effective sphere can be considered as the simplified form factor of the gyroid (*i.e.* the tripod).

Further growth of reduced Au from templated electroless plating was conducted to study the network formation at various stages from the intrinsic tripod through branching. Remarkably, simulations of diffraction results from developing networks as well ordered texture in a specific space group confirm the feasibility of examining the development of network materials through the convolution of the simplified form factor as the building block and the corresponding structure factor with the space-group symmetry.

This methodology should be universal for the examination of the structural evolution of well ordered networks. As a result, the suggested model, by using an effective sphere as the building block and then allocating the sphere at the node positons of the developing network, could be usefully exploited for predicting diffraction results, thus providing new insights into the characteristics of network-structured mater­ials in a specific space group.

## Supplementary Material

Additional information and figures. DOI: 10.1107/S2052252518018389/ti5012sup1.pdf

## References

[bb1] Bates, F. S. & Fredrickson, G. H. (1999). *Phys. Today*, **52**, 32–38.

[bb2] Chu, C. Y., Jiang, X., Jinnai, H., Pei, R. Y., Lin, W. F., Tsai, J. C. & Chen, H. L. (2015). *Soft Matter*, **11**, 1871–1876. 10.1039/c4sm02608j 25635830

[bb3] Crossland, E. J. W., Kamperman, M., Nedelcu, M., Ducati, C., Wiesner, U., Smilgies, D. M., Toombes, G. E. S., Hillmyer, M. A., Ludwigs, S., Steiner, U. & Snaith, H. J. (2009). *Nano Lett.* **9**, 2807–2812. 10.1021/nl803174p 19007289

[bb4] Epps, T. H., Cochran, E. W., Bailey, T. S., Waletzko, R. S., Hardy, C. M. & Bates, F. S. (2004). *Macromolecules*, **37**, 8325–8341.

[bb5] Förster, S., Apostol, L. & Bras, W. (2010). *J. Appl. Cryst.* **43**, 639–646.

[bb6] Hajduk, D. A., Harper, P. E., Gruner, S. M., Honeker, C. C., Kim, G., Thomas, E. L. & Fetters, L. J. (1994). *Macromolecules*, **27**, 4063–4075.

[bb7] Hsueh, H.-Y., Chen, H.-Y., Hung, Y.-C., Ling, Y.-C., Gwo, S. & Ho, R.-M. (2013). *Adv. Mater.* **25**, 1780–1786. 10.1002/adma.201204631 23359456

[bb8] Hsueh, H.-Y., Chen, H.-Y., She, M.-S., Chen, C.-K., Ho, R.-M., Gwo, S., Hasegawa, H. & Thomas, E. L. (2010). *Nano Lett.* **10**, 4994–5000. 10.1021/nl103104w 21047065

[bb9] Hsueh, H.-Y. & Ho, R.-M. (2012). *Langmuir*, **28**, 8518–8529. 10.1021/la3009706 22530553

[bb10] Hsueh, H.-Y., Ling, Y.-C., Wang, H.-F., Chien, L.-Y. C., Hung, Y.-C., Thomas, E. L. & Ho, R.-M. (2014). *Adv. Mater.* **26**, 3225–3229. 10.1002/adma.201305618 24677175

[bb11] Hsueh, H.-Y., Yao, C.-T. & Ho, R.-M. (2015). *Chem. Soc. Rev.* **44**, 1974–2018. 10.1039/c4cs00424h 25622806

[bb12] Hur, K., Francescato, Y., Giannini, V., Maier, S. A., Hennig, R. G. & Wiesner, U. (2011). *Angew. Chem. Int. Ed.* **50**, 11985–11989. 10.1002/anie.201104888 22006868

[bb13] Ichikawa, T., Yoshio, M., Hamasaki, A., Mukai, T., Ohno, H. & Kato, T. (2007). *J. Am. Chem. Soc.* **129**, 10662–10663. 10.1021/ja0740418 17696434

[bb14] Kresge, C., Leonowicz, M., Roth, W. J., Vartuli, J. & Beck, J. (1992). *Nature*, **359**, 710–712.

[bb15] Lin, T.-C., Yang, K.-C., Georgopanos, P., Avgeropoulos, A. & Ho, R.-M. (2017). *Polymer*, **126**, 360–367.

[bb16] Lo, T.-Y., Chao, C.-C., Ho, R.-M., Georgopanos, P., Avgeropoulos, A. & Thomas, E. L. (2013). *Macromolecules*, **46**, 7513–7524.

[bb17] Luzzati, V. & Spegt, P. (1967). *Nature*, **215**, 701–704.

[bb18] Maldovan, M., Urbas, A. M., Yufa, N., Carter, W. C. & Thomas, E. L. (2002). *Phys. Rev. B*, **65**, 165123.

[bb19] Mariani, P., Luzzati, V. & Delacroix, H. (1988). *J. Mol. Biol.* **204**, 165–189. 10.1016/0022-2836(88)90607-9 3216391

[bb20] Matsen, M. W. & Schick, M. (1994). *Phys. Rev. Lett.* **72**, 2660–2663. 10.1103/PhysRevLett.72.2660 10055940

[bb21] Miao, J., Ishikawa, T., Johnson, B., Anderson, E. H., Lai, B. & Hodgson, K. O. (2002). *Phys. Rev. Lett.* **89**, 088303. 10.1103/PhysRevLett.89.088303 12190506

[bb22] Politakos, N., Ntoukas, E., Avgeropoulos, A., Krikorian, V., Pate, B. D., Thomas, E. L. & Hill, R. M. (2009). *J. Polym. Sci. B Polym. Phys.* **47**, 2419–2427.

[bb23] Saranathan, V., Osuji, C. O., Mochrie, S. G. J., Noh, H., Narayanan, S., Sandy, A., Dufresne, E. R. & Prum, R. O. (2010). *Proc. Natl Acad. Sci. USA*, **107**, 11676–11681. 10.1073/pnas.0909616107 PMC2900708 20547870

[bb24] Scherer, M. R., Li, L., Cunha, P., Scherman, O. A. & Steiner, U. (2012). *Adv. Mater.* **24**, 1217–1221. 10.1002/adma.201104272 22287187

[bb25] Seddon, J. M. & Templer, R. H. (1993). *Philos. Trans. R. Soc. Lond. A*, **344**, 377–401.

[bb26] Takenaka, M., Wakada, T., Akasaka, S., Nishitsuji, S., Saijo, K., Shimizu, H., Kim, M. I. & Hasegawa, H. (2007). *Macromolecules*, **40**, 4399–4402.

[bb27] Wilts, B. D., Michielsen, K., Kuipers, J., De Raedt, H. & Stavenga, D. G. (2012). *Proc. R. Soc. B Biol. Sci.* **279**, 20112651. 10.1098/rspb.2011.2651 PMC3350696 22378806

[bb28] Yu, K., Fan, T., Lou, S. & Zhang, D. (2013). *Prog. Mater. Sci.* **58**, 825–873.

[bb29] Zeng, X., Ungar, G. & Impéror-Clerc, M. (2005). *Nat. Mater.* **4**, 562–567. 10.1038/nmat1413 15937487

