# Supplementary material for: Examination of well ordered nanonetwork materials by real- and reciprocal-space imaging
Source: IUCrJ. 2019 Feb 15;6(Pt 2):259–66. doi: 10.1107/S2052252518018389 (PMC6400199; doi:10.1107/S2052252518018389)
Supplement: Supplementary file 1 [file m-06-00259-sup1.pdf]

IUCrJ

Volume 6 (2019)

Supporting information for article:

Examination of well ordered nanonetwork materials by real- and reciprocal-space imaging

Po-Ting Chiu, Yu-Cheng Chien, Prokopios Georgopanos, Ya-Sen Sun, Apostolos Avgeropoulos and Rong-Ming Ho

We provide here some further information about the characterization of the templates we used: the details of thermal treatment before collecting SAXS data, the discussion of fitting using a cylindrical model and the structural evolution of the template with large dimensions.

S1. Characterization of templates

The PS-PDMS samples were synthesized by Avgeropoulos *et al*. (Politakos *et al.*, 2009) The templates were fabricated through solvent casting in toluene followed by HF acid etching.

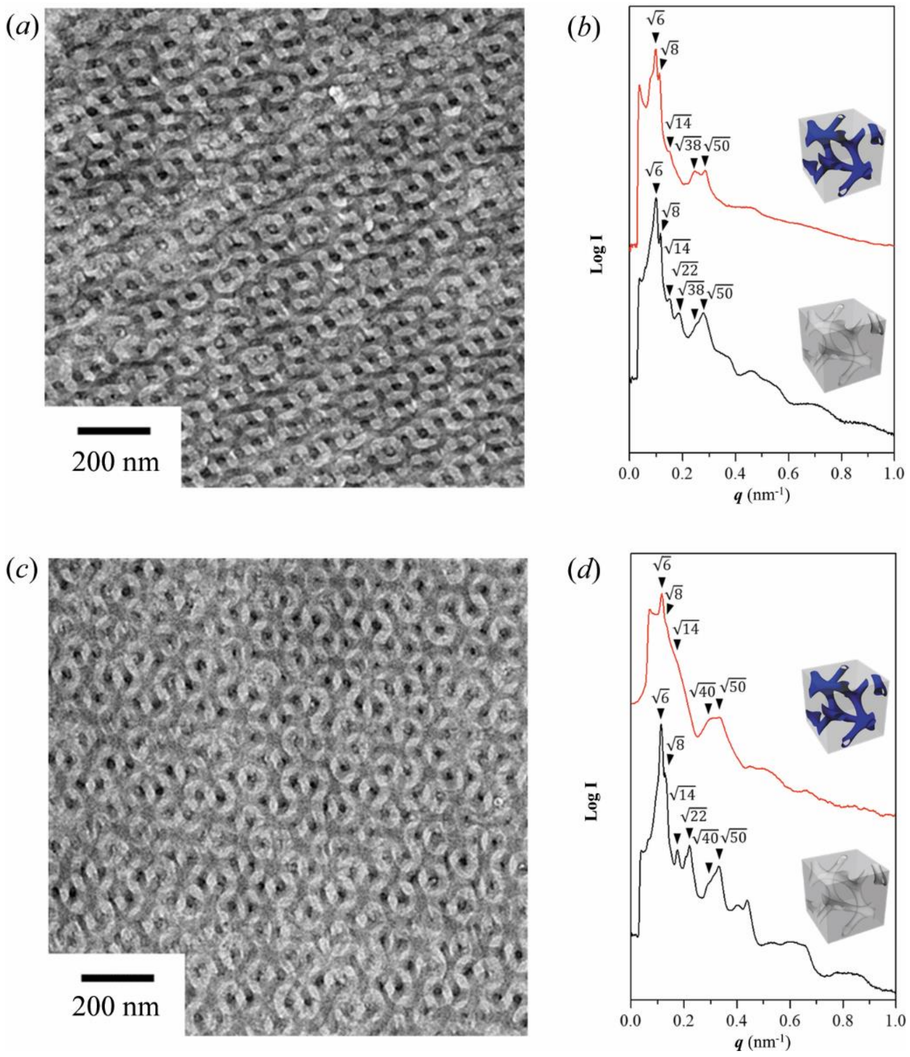

Figure S1 TEM [110] projections and one-dimensional SAXS profiles of self-assembled PS-PDMS with (A, B) large molecular weight and (C, D) small molecular weight after casting from toluene and the corresponding porous PS template after etching of PDMS block.

S2. SAXS results and fittings

As shown in **Figure S2A**, the scattering from nanoporous template can be removed after the collapse of gyroid structured nanochannel. Even with Au nanoparticles inside (**Figure S2B**), the reflections can be eliminated once the temperature is higher than the glass transition temperature of PS.

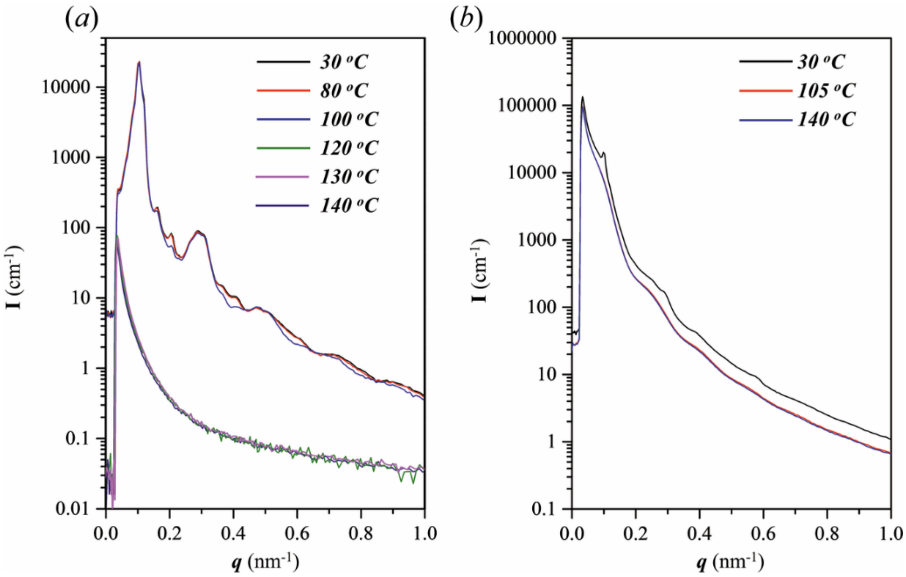

Figure S2 Temperature-resolved *in-situ* SAXS results of (A) neat PS template and (B) PS/Au nanohybrids during thermal treatment.

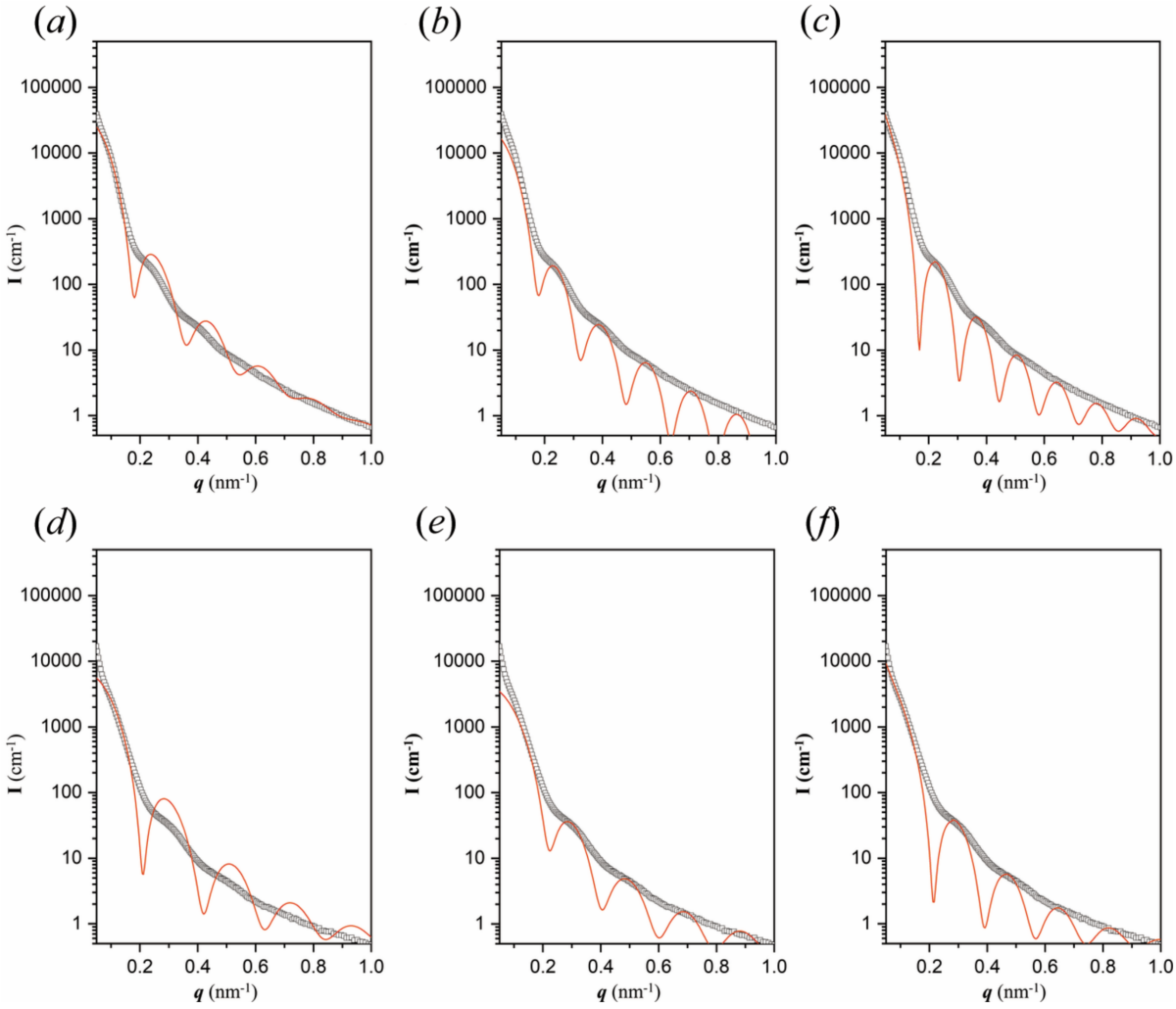

Figure S3 Fittings of SAXS profiles of reduced Au nanoparticle using (A,D) disk, (B,E) cylinder and (C,F) cube models for (A-C) large-sized and (D-F) small-sized tripod Au fabricated. The thickness and radius of disk in (A) are 35.4 nm and 13.5 nm; the length of cube in (B) is 40.0 nm; the radius and length of cylinder in (C) are 23.0 nm and 174.0 nm. The thickness and radius of disk in (D) are 15.0 nm and 10.2 nm; the length of cube in (E) is 32.0 nm; the radius and length of cylinder in (F) are 18.0 nm and 96.8 nm

S3. Structural evolution of large-sized Au

As shown in **Figure S4A**, branched Au with approximately 100 nm in effective diameter can be found after two-day growth. With the continuous growth, the size of branched Au becomes larger (**Figure S4B**). Consistently, the branched Au tripod develops as an ordered network with spherical shape and the diameter is approximately 400 nm which is about two to three unit cells (**Figure S4C**). After six-day growth from templated electroless plating, the size of networked Au tripod which can reach 600 nm, is approximately six unit cells (**Figure S4D**). Consequently, it is expected to see the relative *q* values of $\sqrt{2}:\sqrt{6}:\sqrt{8}$ for the presence of $I4_132$ symmetry from the reciprocal-space imaging. However, there are no significant reflection peaks with two-day growth (**Figure S4E**). Even at the later stages, the characteristic reflections of gyroid are not as evident as expected to be distinguished from the profile of form factor (**Figures S4F-S4H**). The unexpected scattering results are attributed to the limited growth on grain size from the template with large pore size. The FWHM in **Figure S4E** is so broad so that, based on the concept of Scherrer equation for the estimated grain size, it should be too small to reach the required grain size for significant reflections from the developed symmetry. Note that the FWHM can be gradually reduced following the subsequent growth, but it is not able to reach the required size for the expected reflections (**Figures S4F-S4H**). To attain the characteristic reflections of the forming gyroid, larger grain size is demanded. Yet, it is too time-consuming for the further growth of Au with the large template.

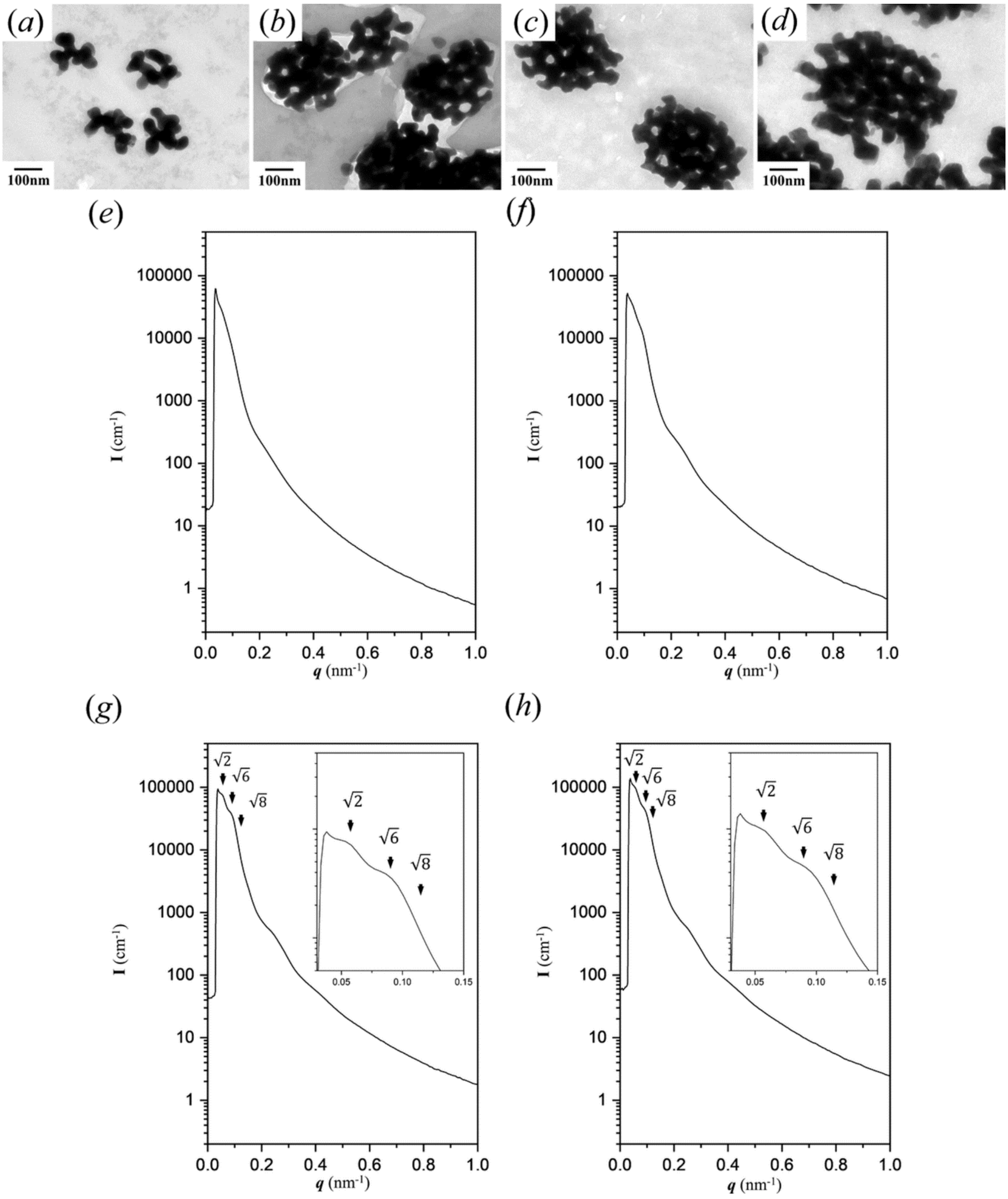

Figure S4 TEM, SEM images and SAXS profiles of gyroid Au nanoparticle with large dimension at late stages with templated electroless plating for (A, E, I) 2 day, (B, F, J) 3 day, (C, G, K) 5 day and (D, H, L) 6 day. The characteristic reflections of gyroid with relative q values of $\sqrt{2}$: $\sqrt{6}$: $\sqrt{8}$ are marked by the triangles. The insets are the enlarged low q region.

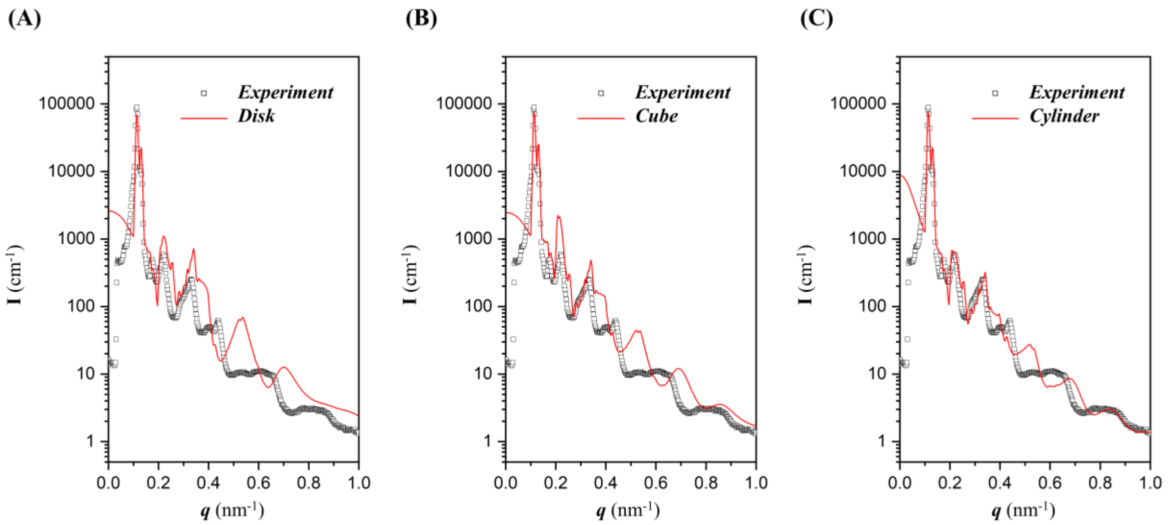

Figure S5 Simulations of SAXS results from template with small-sized gyroid texture using form factors of (A) disk, (B) cube and (C) cylinder. The structure factor with lattice parameter of 135.2 nm was used based on the SAXS result.
